# Using the Nutri-Score to visualise food reformulation in Germany: the case of breakfast cereals

**DOI:** 10.1186/s12889-024-21102-7

**Published:** 2025-01-04

**Authors:** Corinna Gréa, Anna Dittmann, David Wolff, Hanna Haidar, Silvia Roser, Benedikt Merz, Stefan Storcksdieck genannt Bonsmann

**Affiliations:** 1 Department of Nutritional Behaviour, Max Rubner-Institut (MRI) – Federal Research Institute of Nutrition and Food, Haid-und-Neu-Straße 9, 76131 Karlsruhe, Germany; 2 Department of Physiology and Biochemistry of Nutrition, Max Rubner-Institut (MRI) – Federal Research Institute of Nutrition and Food, Haid-und-Neu-Straße 9, 76131 Karlsruhe, Germany; 3 Presidential Office, Max Rubner-Institut (MRI) – Federal Research Institute of Nutrition and Food, Haid-und-Neu-Straße 9, 76131 Karlsruhe, Germany

**Keywords:** Breakfast cereals, Reformulation, Nutrition labelling, Nutri-Score, Children’s products, Sugar reduction, Germany, Monitoring, Packaged food

## Abstract

**Background:**

The reformulation of commonly consumed foods towards less sugar, fat, and salt is an important public health strategy to improve food choices of consumers and thus address the high prevalence of overweight and obesity. Front-of-pack nutrition labels like the Nutri-Score may drive reformulation and support nutritionally favourable food choices. Breakfast cereals are of special interest in that they tend to be high in sugar and are relatively often targeted at children. This study therefore aimed to evaluate the German breakfast cereal market in terms of reformulation efforts and to what extent these would show in a better Nutri-Score.

**Methods:**

Using data from the German monitoring of packaged foods, changes in energy and nutrient content and the final nutritional score (FNS) of breakfast cereals, differentiated into children’s and non-children’s products, between 2019 (*n* = 888) and 2022 (*n* = 1473) were evaluated (Mann-Whitney test for two independent samples). Reformulation efforts were analysed in a subsample of paired products available in both years (*n* = 424). The Nutri-Score was calculated using the 2023 algorithm.

**Results:**

Sugar content of children’s and non-children’s breakfast cereals decreased by 25.5% (*p* < 0.001) and 8.7% (*p* < 0.001), respectively, while fat content increased (+ 32.0% (*p* < 0.001) and + 7.0% (*p* < 0.036)). Especially for children’s breakfast cereals, the share of products with a ‘green’ (A or B) Nutri-Score was higher in 2022 than in 2019. At the same time, the share of less favourable breakfast cereals (Nutri-Score C-E) was higher for children’s than for non-children’s breakfast cereals in both years. For paired products, the FNS changed (positively or negatively) in 34.6% and concomitantly the Nutri-Score in 14.2% of cases. Products showing a better Nutri-Score were often reformulated in a way to just make it into the better class.

**Conclusion:**

Improvements in nutrient content and FNS of breakfast cereals in Germany were mainly driven by shifts in the product portfolio, not by reformulation of existing products. Hence, any benefit for public health would require that consumers switch to (newly introduced) breakfast cereals with a more favourable composition. Overall, more reformulation efforts guided by a holistic product monitoring are needed to improve the nutritional quality of the food supply.

**Supplementary Information:**

The online version contains supplementary material available at 10.1186/s12889-024-21102-7.

## Background

More than half of the adult population in Germany is overweight, about 20% obese [1, 2]. Corresponding figures for children are 15% and 6%, respectively [3]. Improving diet quality is an important means of tackling this growing health problem. Nutrition policies such as the reformulation of packaged food as well as the use of front-of-pack nutrition labels (FoPLs) are seen as effective interventions to create a healthier food environment and thus improve population diets [4, 5].

The World Health Organization defines food reformulation as “altering the processing or composition of a food or beverage product, to improve its nutritional composition or to reduce its content of ingredients or nutrients of concern” [5]. In 2016, the European Union urged Member States to implement corresponding reformulation strategies [6]. In response, Germany initiated the “National Reduction and Innovation Strategy for Sugar, Fats, and Salt in Processed Food” (NRI), which is based on voluntary commitments by the industry and accompanied by an annual product monitoring [7]. One voluntary commitment by the food sector concerns breakfast cereals and aims to reduce the sugar content of products targeted at children by at least 20% until the end of 2025 [8]. Breakfast cereals is one of the food categories with a high customer reach, especially popular among children, and extensively marketed to them [9, 10], they are a prioritised food group in reformulation strategies of various countries [e. g. 11–14].

Although reformulation of packaged food can help improve the nutritional quality of food and enable individuals to consume a healthier diet to a certain degree [5], nutritionally favourable options are often not easily recognisable for consumers. Here, the role of FoPLs as consumer guidance comes into play [15]. Among FoPLs, the Nutri-Score as a colour-coded, evaluative FoPL is an efficient tool to facilitate comparisons between products within a food category, enabling consumers to make informed and potentially nutritionally favourable food choices [16–19].

Developed and first introduced in France in 2017, the Nutri-Score has now been adopted as the official FoPL in seven European countries, including Germany in 2020. A proposal for a harmonised mandatory FoPL at EU level, as part of the Farm to Fork strategy, is currently pending, with the Nutri-Score being one of the potential labels discussed [20]. Although the use of the Nutri-Score is so far voluntary, food manufacturers or retailers that choose to use the Nutri-Score are obliged in general to display the FoPL on all their products within 2 years [21]. The Nutri-Score undergoes regular revisions, including the most recent one in 2023, with the consistent goal to improve the discrimination between products with high and low contents of unfavourable nutrients. Smaller increments in the point allocation scale for instance for sugar allow for a stricter and therefore more adequate Nutri-Score rating. As the discrimination of sugary products was considered a key focus in this context, breakfast cereals were included as a priority product group [22, 23]. In Germany, the 2023 algorithm was introduced in January 2024, with a 24-month transition period [22].

With regard to current national reformulation efforts within the NRI, this paper aims to answer the following research questions:

1. Are there changes in the energy and nutrient content of breakfast cereals between 2019 and 2022 on the German market?
2. Did producers reformulate existing products within this time frame?
3. If products have been specifically reformulated, does this affect the Nutri-Score classification?

To this end, based on data from the German monitoring of packaged food [24], changes in energy and nutrient contents of breakfast cereals targeted at children (children’s breakfast cereals) and breakfast cereals not targeted at children (non-children’s breakfast cereals) on the German market between 2019 and 2022 were evaluated. To determine whether the nutrient composition of individual breakfast cereals had changed, a sample of paired products that were available at both time points of data collection was analysed. Based on this subsample, it was assessed which of the observed changes in the nutrient composition affected the Nutri-Score classification.

## Methods

### Data collection and management

Data on breakfast cereals were gathered from August to December in 2019 and 2022, respectively, as part of the German monitoring of packaged food. Data collection comprised the energy and nutrient contents as stated in the mandatory nutrition declaration (fat, saturated fat, carbohydrate, sugar, protein, and salt) [25]. Data on fibre was recorded when provided. Where available, additional labelling information such as ingredient lists, label on organic production or Global Trade Item Number (GTIN) was collected. In order to cover the market as broadly as possible, data was predominantly collected online via the manufacturers’ websites. To fill data gaps, the online research was complemented by enquiries with manufacturers and visits to grocery stores. The data were managed with a customised branded food module within the FoodCASE software, version 7.9.1 (Premotec GmbH, Winterthur, Switzerland). In the case of implausible values or missing data concerning energy or nutrient content, manufacturers were contacted to verify, correct, or complete the respective information. In case of no answer, these products were excluded from analysis. Detailed information on the design and methods of the German monitoring of packaged food is published elsewhere [7].

#### Inclusion criteria and definition of food categories

Breakfast cereals for which the nutritional information was available for the unprepared product, i.e. without the addition of milk or other components, were considered. Plain rolled grains without any other ingredients and savoury porridges (e.g. tomato-broccoli porridge) were excluded. Furthermore, breakfast cereals consisting mainly of nuts and seeds rather than cereals and declared as “protein”, “power”, or “sport” were eliminated, as a significantly different nutritional composition was expected. All included products were grouped into one of three categories (see Table 1) based on the product name and/or ingredient list.

**Table 1:** Description of breakfast cereal categories

Category	Definition	Examples
Muesli and porridge	Contain quick cooking or rolled cereal grains, pseudo cereals, or soya flakes as main ingredients. May contain dried fruits, nuts, chocolate, or other ingredients.	Crunchy nut and fruit muesli; apple cinnamon porridge
Flakes	Made from corn, oats, rice, or other cereal grains. Not containing any fruits, nuts or chocolate, except for in the coating.	Cornflakes; frosted flakes
Other cereal products	Made from toasted, puffed or popped cereal grains shaped into biscuits, as well as flakes with additional ingredients such as fruits, nuts or chocolate. May contain chocolate or cocoa.	Nougat bits; cinnamon chips; choco balls

Given the importance of breakfast cereals in children’s diets, a distinction was made between children’s breakfast cereals and those not targeted at them, in order to look separately at the nutrient composition and product reformulation. Within the three categories, the differentiation was based on the design of the packaging or the product itself. Table 2 describes the four criteria, of which a given product must fulfil at least one in order to be classified as children’s breakfast cereal.

**Table 2:** Criteria for products classified as “children’s”

Criteria	Examples
The product name includes “child(ren)” or “kids” etc. or appeals directly to children	“Chocolate Bears”
The packaging is attractively designed for children	Displaying smiling animals or cartoon characters
The food product itself or its components is/are designed for children	Cereals in the shape of bears or letters
The packaging includes information aimed at parents or children	“for your little ones”, notes on games for children, learning effects, or information about free toys or collectible picture cards inside

#### Product pairing

In order to assess whether the nutrient composition of a given breakfast cereal changed over time, a subsample of breakfast cereals present on the market in both survey years (2019 and 2022) was analysed. Such pairing of products over time is not performed in the German monitoring of packaged food but was carried out as part of the EU Joint Action Best-ReMaP, in which Germany took part [14]. Pairing was done manually as follows: (1) GTIN for each product of 2022 was used to identify a matching GTIN in the 2019 data. (2) if there was no result of matching GTINs or no GTIN available, product information, including brand name, product name, legal name, flavour, and net weight were used to search for a match. The ingredient list and the nutritional values could be different, but product or legal name had to be the same or close. If two products of 2022 could be assigned to one product of 2019, the product nearest in net weight was counted as a match. Breakfast cereals that had changed the design over the years, resulting in a different classification of a product as child-targeting or not in 2019 and 2022, were classified according to the 2022 design for the subsample of paired products.

#### Calculation of the Nutri-Score

The 2023 algorithm of the Nutri-Score for general foods [22, 23] was used to compute the overall nutritional value of breakfast cereals (years 2019 and 2022). In brief, ‘unfavourable’ points were allocated for energy (0 to 10 points), saturated fats (0 to 10 points), sugar (0 to 15 points), and salt (0 to 20 points), whereas ‘favourable’ points were allocated to proteins (0 to 7 points) as proxy for iron and calcium, fibres (0 to 5 points), and the percentage of fruits, vegetables, and legumes (henceforth described as F&V component) (0 to 5 points). The ‘unfavourable’ (0 to 55 points in total) and ‘favourable’ points (0 to 17 points in total) are balanced out in the computation of the final nutritional score (FNS). However, ‘favourable’ points for protein are disregarded, if a product scores more than 11 ‘unfavourable’ points. In consideration of this rule, the FNS may range from − 17 (best rating) to 45 points (worst rating). The FNS is attributed a Nutri-Score ranging from A to E (Table 3). In our study, products classified as C, D, or E (FNS ≥ 3) are defined as ‘less favourable’. The computation of the Nutri-Score is based on the declared nutritional values per 100 g for energy, saturated fats, sugar, protein, salt, and fibre. Based on the ingredient list, the percentage of F&V was estimated by an experienced nutritionist using the Nutri-Score FAQ document [26]. If the ingredient list was not available on the manufacturer’s website or fibre content was not declared, the product was excluded.

**Table 3:**
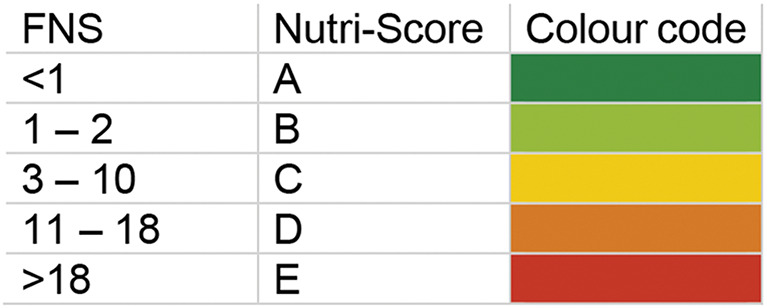
Final nutritional score (FNS) ranges with their corresponding Nutri-Score classification and colour code

### Statistical analysis

All statistical analyses were performed using the software R, version 4.3.2. The normality of the data distribution was tested using the Shapiro-Wilk test and rejected. Consequently, the non-parametric Mann-Whitney test for two independent samples was used to compare energy and nutrient contents as well as the FNS of children’s breakfast cereals respectively non-children’s breakfast cereals between survey years 2019 and 2022. For all results, p-values < 0.05 were considered as statistically significant. Furthermore, shares of pairs that showed a change in FNS were calculated. This subsample is further analysed as the individual scores of the FNS in 2019 and 2022 were plotted against each other and the effects on the Nutri-Score classification described.

## Results

### Changes in the nutritional composition of the breakfast cereal offer on the German market

**Fig. 1:**
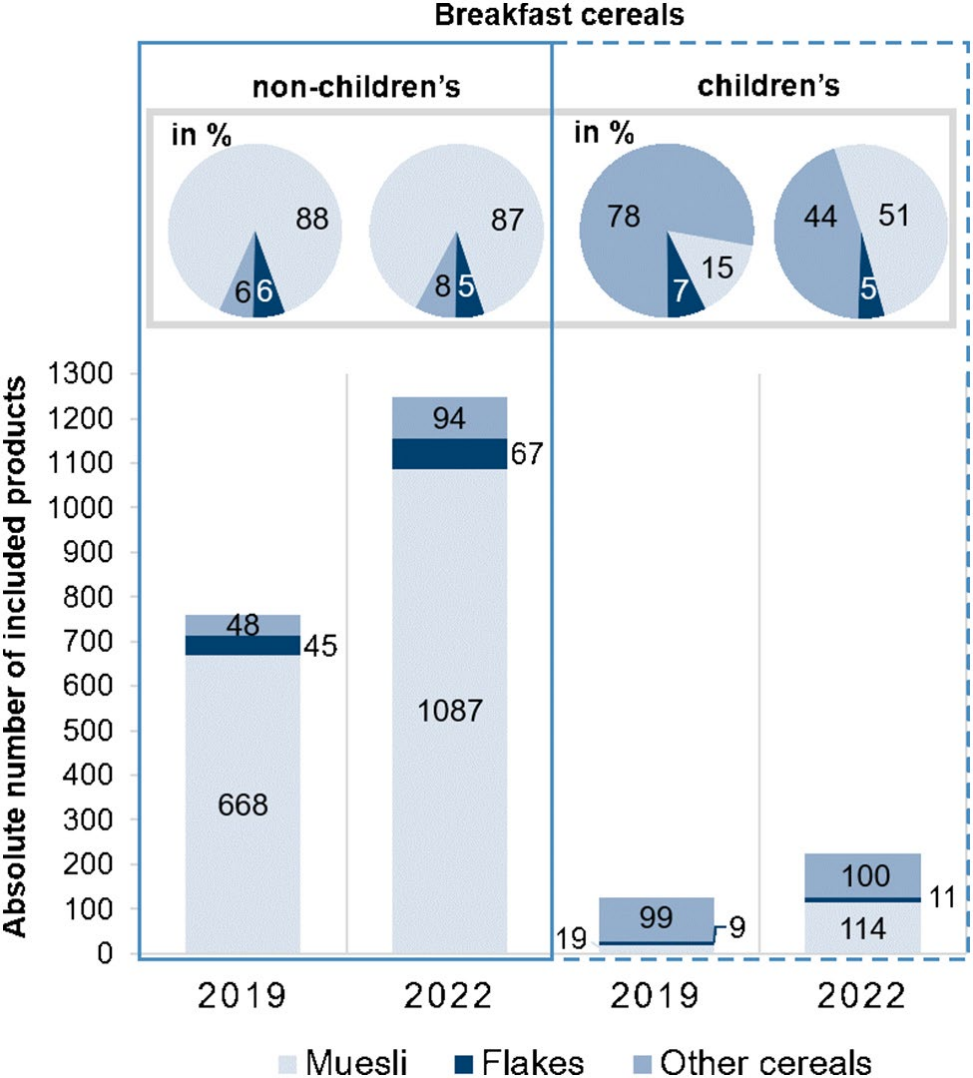
Distribution across the subcategories differentiated into non-children’s and children’s breakfast cereals for data collection years 2019 and 2022

A total of 888 and 1473 breakfast cereals were included in the sample of 2019 and 2022, respectively. Overall, 14% of the breakfast cereals in 2019 (*n* = 127) and 15% of the breakfast cereals in 2022 (*n* = 225) were classified as children’s breakfast cereals. Figure 1 shows that in particular more mueslis and porridges (hereafter referred to as muesli) have been recorded over the years. However, only for children’s breakfast cereals, the distribution across the categories shifted clearly to muesli. The category of flakes constituted the smallest group in each year.

**Fig. 2:**
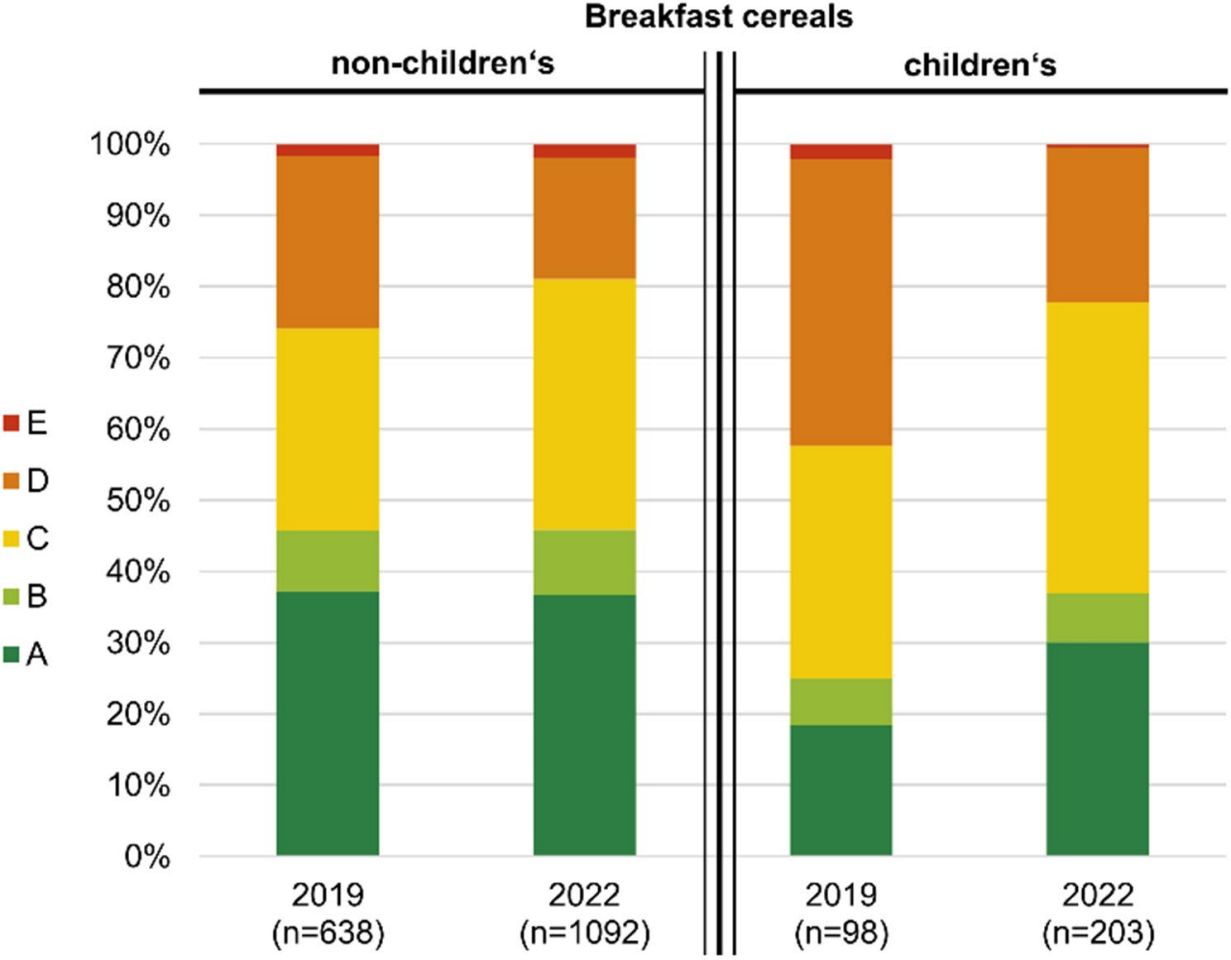
Shares of Nutri-Score classification differentiated into non-children’s and children’s breakfast cereals in Germany for data collection years 2019 (*n* = 736) and 2022 (*n* = 1295)

The comparison between the subsamples revealed significantly lower sugar and significantly higher fat contents in 2022 than in 2019. Children’s breakfast cereals demonstrated a greater percentage change, with a 25.5% reduction in sugar (*p* < 0.001) and a 32.0% increase in fat (*p* < 0.001) than non-children’s breakfast cereals with − 8.7% (*p* < 0.001) and + 7.0% (*p* = 0.036), respectively. For energy, no significant change was found (*p* = 0.913; *p* = 0.844). Overall, mean FNS was only significantly reduced in children’s breakfast cereals, by 3 points (*p* < 0.001) (see Table 4). The share of products with a ‘green’ (A or B) classification as well as products classified as Nutri-Score C was higher in 2022 than in 2019. At the same time, the share of Nutri-score D products was lower. The share of breakfast cereals classified as less favourable (Nutri-Score C-E) was higher for children’s breakfast cereals (see Fig. 2). At category level of children’s breakfast cereals, a reduction of sugar content was found in other cereal products (12.4%, *p* = 0.003) and an increase of fat content in muesli (39.0%, *p* = 0.016) and flakes (149.3%, *p* = 0.016). For non-children’s breakfast cereals, a reduction of sugar content was shown in muesli (9.2%, *p* < 0.001) and flakes (25.9%, *p* = 0.032). The latter also showed an increase in fat content (99.5%, *p* = 0.031). For details on changes at category level, see Supplement File 1.

**Table 4:** Mean energy and nutrient contents of breakfast cereals surveyed in Germany in 2019 (*n* = 888) and corresponding changes in 2022 (*n* = 1473)

	Breakfast cereals							
	Non-children‘s				Children‘s			
	mean in 2019 (*n* = 761)	mean change in 2022 (*n* = 1248)			mean in 2019 (*n* = 127)	mean change in 2022 (*n* = 225)		
		abs.	rel. (%)	sig.^1^		abs.	rel. (%)	sig.^1^
Energy [kcal/100 g]	400.7	+ 0.8	+ 0.2		390.3	+ 1.0	+ 0.3	
Fat [g/100 g]	10.7	+ 0.7	+ 7.0	*	5.5	+ 1.8	+32.0	***
Saturated Fat [g/100 g]	3.1	0.0	+ 0.5		1.6	+ 0.5	+33.7	**
Carbohydrate [g/100 g]	60.9	-2.5	-4.1	***	73.9	-5.6	-7.6	***
Sugar [g/100 g]	15.7	-1.4	-8.7	***	22.9	-5.8	-25.5	***
Protein [g/100 g]	10.9	+ 1.0	+ 8.9	***	8.4	+ 1.2	+14.0	***
Salt [g/100 g]	0.25	-0.03	14.1	**	0.43	-0.20	-45.5	***
Fibre [g/100 g]^2^	8.8	+ 0.4	+ 4.1	*	6.1	+ 1.4	+22.8	***
Final nutritional score^3^ (FNS)	4.7	-0.3	-5.5		8.7	-3.1	-35.9	***

^1^Statistically significant change in mean content in 2022 compared to 2019 (*p < 0.05; **p < 0.01; ***p < 0.001)

^2^Sample size differs, as the fibre content was not available for 123 (2019) resp. 154 (2022) non-children’s breakfast cereals and 29 (2019) resp. 22 (2022) children’s breakfast cereals

^3^Sample size differs, as the fibre content and/or ingredient list was not available to calculate the FNS for 123 (2019) resp. 156 (2022) non-children’s breakfast cereals and 29 (2019) resp. 22 (2022) children’s breakfast cereals

### Reformulation of breakfast cereals

In order to examine whether a given product underwent reformulation, the FNS and Nutri-Score of a subsample of paired breakfast cereals (*n* = 424), i.e. marketed under the same name in 2019 and 2022, was further analysed.

A change in the FNS occurred in 34.6% of all pairs (Table 5). The share of pairs showing an improved nutritional value was higher than the share of pairs which deteriorated. For children’s breakfast cereals the share of pairs that deteriorated was higher compared to non-children’s breakfast cereals (20.0% vs. 9.9%).

**Table 5:** Changes of the final nutritional score (FNS) and Nutri-Score for all pairs of breakfast cereals on the German market in 2019 and 2022 stratified into non-children’s and children’s products

	Pairs *n* (%)	FNS change *n* (%)	More favourable	Less favourable	Nutri-Score change *n* (%)	More favourable	Less favourable
All pairs	424 (100%)	147 (34.6%)	99 (23.3%)	48 (11.3%)	60 (14.2%)	44 (10.4%)	16 (3.8%)
Non-children’s	364 (100%)	119 (32.7%)	83 (22.8%)	36 (9.9%)	46 (12.6%)	36 (9.9%)	10 (2.7%)
Children’s	60 (100%)	28 (46.7%)	16 (26.7%)	12 (20.0%)	14 (23.3%)	8 (13.3%)	6 (10.0%)

### Effects of reformulated breakfast cereals on the Nutri-Score classification

**Fig. 3:**
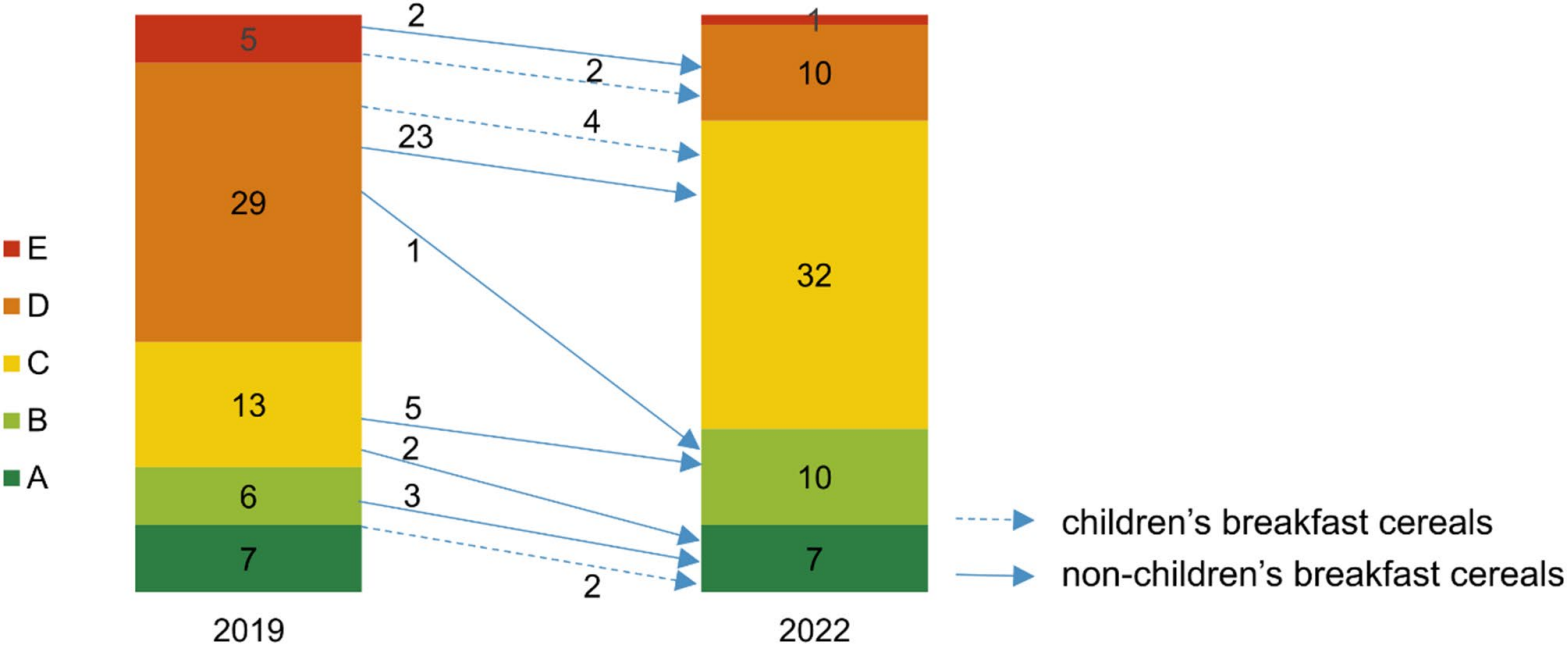
Shifts in Nutri-Score classification of pairs showing a change (*n* = 60); arrows indicate an improvement

**Fig. 4:**
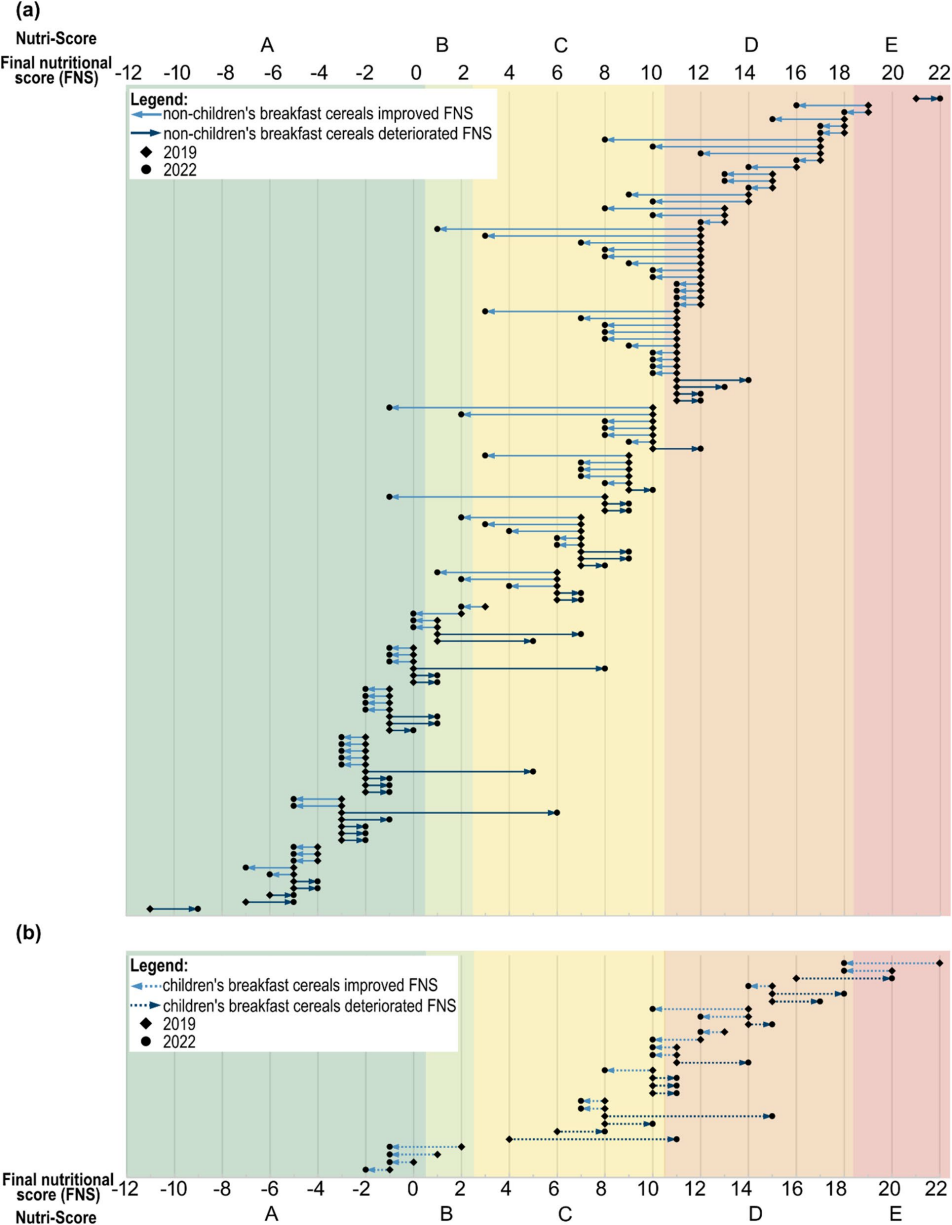
Final nutritional score (FNS) and corresponding Nutri-Scores for (**a**) all non-children’s and (**b**) all children’s pairs of breakfast cereal products with a change in FNS (*n* = 147). Sorted in ascending order by FNS of 2019 (complete FNS scale ranges from − 17 to 45 points)

Only for a small number of pairs (60 out of 424 products) did reformulation have an effect on the Nutri-Score classification (Table 5). Of these, most products (45%; *n* = 27) shifted from Nutri-Score D to Nutri-Score C (Fig. 3). In the case of children’s breakfast cereals, these were exclusively products belonging to the category of other cereal products, while for non-children’s breakfast cereals these were mainly flakes and muesli products. Only few products (13%; *n* = 8) improved their rating to ‘green’ (Nutri-Score A or B) (especially mueslis) (Fig. 3). In 27% of products (*n* = 16), the Nutri-Score classification deteriorated, with shifts mostly from class A to B or C (only non-children’s products) or from C to D (only children’s products) (Fig. 4).

Changes in the FNS that led to a change in the Nutri-Score classification were in the range of -11 to -1 (improvement) and in the range of + 1 to + 9 (deterioration) points, respectively. The most often observed changes in FNS were in the range of -1 to -4 points. Most products achieved an improvement in FNS by reducing sugar (-0.1 to -10 g/100 g), often accompanied by FNS-affecting changes in fibre (+ 0.1 to + 3.0 g/100 g), protein (+ 0.1 to + 3.6 g/100 g), saturated fat (-0.1 to -5.2 g/100 g), or salt (-0.09 to -1.3 g/100 g), or a combination of these. The largest FNS improvements were primarily driven by reducing saturated fat, often by substituting palm oil with sunflower oil, or by reducing salt content. For most products with a reduction of at least 5 FNS points, the number of ‘unfavourable‘ points was below the limit of 11 points, resulting in considering the ‘favourable‘ points for protein content in the product of 2022, which were not counted in the product of 2019.

For products with a large increase in FNS (deterioration), increases in sugar content (+ 4 to + 6.6 g/100 g) and saturated fat content (+ 0.1 to + 4.4 g/100 g) in particular forced the disregard of the positive points for the protein content.

As Fig. 4 illustrates, not only the magnitude of the change in the FNS score was decisive for a change in Nutri-Score classification, but also the initial position of a product (whether it was close to a class threshold or not) as well as the initial class itself. Since many of the products in category D were close to the threshold to category C, relatively small changes of 1–2 FNS points were already sufficient to change the Nutri-Score classification to C.

## Discussion

This study investigated the nutrient composition of children’s and non-children’s breakfast cereals on the German market in 2019 and 2022, particularly with regard to reformulation and the potential of the Nutri-Score to visualise such.

### Developments on the breakfast cereal market in Germany

Our sample indicated that, between 2019 and 2022, the number of breakfast cereal products on the German market grew substantially, with concomitant changes in the average nutrient content. Whereas the average sugar content decreased significantly, especially in the group of children’s breakfast cereals, the average fat content increased significantly. Our results are in line with findings from other countries that showed declining sugar contents in this product group in the period between 2004 and 2020 [27–29]. A concomitant significant increase in average fat content as in our study was only found for the Australian market (period 2013 to 2020) in different subcategories (granola and clusters, hot flavoured and plain cereal (porridge), and muesli) as well as overall in a sample of paired products [27]. Our results on subcategory level showed these concomitant changes only for flakes. This diverse subcategory ranges from plain, unsweetened flakes to sugar- or chocolate-coated flakes high in sugar and fat, respectively. Whether this interaction (fat up, sugar down) shown for the overall non-paired sample is also evident on the single product level was outside the scope of this study but changes in Nutri-Score could help answer this question. A first look at the subsample of paired breakfast cereals with changes in Nutri-Score due to a decrease of negative points by a sugar reduction (e.g. -8.0 g/100 g) indicates that this interaction exists, although the increase in fat was rather small (e.g. 0.5 g/100 g).

Pairing the products from different time points revealed a fast-changing breakfast cereal market in Germany: out of 888 products in 2019, only 424 (47.7%) were still on the market in 2022. The product monitoring in France specifically analyses market changes and also shows that the overall market is mainly influenced by the introduction of new products and products that are withdrawn from the market. Between 2011 and 2018, 63% of the sample were newly introduced products [30]. Therefore, the observed changes in average nutrient content are explainable by shifts in the portfolio of products over the years rather than the reformulation of existing products. Compared to 2019, the 2022 subsample contained substantially more mueslis (especially porridges), and these typically have lower sugar content but higher fat content than other categories [24]. On the one hand, these market developments do appear beneficial for public health in terms of improving the food offer, as many mueslis in particular had come onto the market, many of which have a nutritionally favourable composition. Increasing demand for alternatives to classic cereals such as froot loops, honey pops or chocolate crispies (other cereals category), as well as more opportunities for product variation in the muesli category, could be the reason for the growing muesli market, which has had a major impact among children’s cereals in particular. This is supported by a pronounced shift in consumption shares away from other cereals towards muesli, but also rolled oats and cornflakes, in 2021 [31].

### Visualisation of food reformulation

FoPL is discussed as a lever for reformulation and thus for improving the product composition [28, 32]. However, our findings for paired products and based on the Nutri-Score algorithm as updated in 2023 demonstrate that little progress has been made regarding the improvement of the overall nutritional composition on the single product level. Only for a few products did reformulation have an impact on the Nutri-Score classification; this observation may have been different with the more lenient 2015 algorithm in force in the survey years. Particularly in the case of children’s products, a comparatively large share has actually deteriorated in the Nutri-Score. While this may be an artefact from using the stricter 2023 algorithm, it emphasises the gap between actual product formulation and what would be desirable in terms of public health.

Moreover, if a product had been reformulated, the product often fell just below the threshold of the next better Nutri-Score class, which is particularly noticeable in children’s products. One might argue that this again could be an artefact from using the stricter 2023 algorithm of the Nutri-Score, when the 2015 algorithm was the point of reference in 2019 and 2022. However, our observation is paralleled by recent results for the French market and based on the 2015 algorithm: even though the introduction of the Nutri-Score brought breakfast cereals with a more favourable nutritional composition to the market, an accumulation of products just below the threshold of the next better Nutri-Score class was recognised [33]. Hence, products are not necessarily reformulated in a way that would make their being nutritionally favourable visible through a better Nutri-Score. Where improvements in Nutri-Score emerged, this was explained largely by decreases in sugar and salt content and increases in fibre and protein content [33]. Our study extends these findings by exploring how changes in the individual nutrients interrelate to improve the Nutri-Score classification in the end. We found that the improvement was primarily due to a combination of sugar reduction and increase in protein or fibre content. Our analyses also revealed that the number of negative points can be considerably reduced, particularly by lowering saturated fat, as the range between two thresholds (1 g/100 g per level) are comparatively lower than for sugar (4.5 g/100 g per level). In this regard, substituting palm oil through e.g. sunflower oil, seems to be an easy leverage point for mueslis in particular to improve their overall rating, as we could determine for some crunchy mueslis. Further modelling studies at the individual food level are necessary to provide information as to what compositional changes would be the most feasible in order to achieve a better overall Nutri-Score.

Although we were able to demonstrate that reformulation within breakfast cereals could be visualised via the Nutri-Score and thus can be an opportunity for food producers to positively highlight their own products in comparison to competing products receiving a less favourable rating, the visibility for consumers appears to remain limited so far. As per current EU regulation [25], usage of the Nutri-Score is and can only be voluntary. A first survey, conducted just after the implementation of the Nutri-Score in Germany, showed a labelling of less than 10% of the included cereals [34]. A snapshot taken about a year later noted a coverage of 28% [35]. Despite this increase, the data suggest that the vast majority of breakfast cereals on the German market do not carry a Nutri-Score. Unless the Nutri-Score is applied on all products, the empowerment of consumers to quickly identify nutritionally favourable products within a given food category remains impaired. Food manufacturers could send a strong signal of consumer support by adopting the Nutri-Score as the government-endorsed FoPL in Germany on all their products. This is supported by comprehensive evidence reviews concluding that summary indicators with or without colour-coding help consumers best in identifying and choosing the nutritionally favourable option [16, 36, 37]. It must be borne in mind though that the Nutri-Score as a FoPL should not be used as an overall guidance on ‘healthy’ products.

### Reformulation policies

Food reformulation is often viewed as one of several means to reduce the prevalence of overweight and obesity. However, as observed in our data and already discussed, measures targeting only individual nutrients (e.g. sugar), can lead in some product groups to an increase in another nutrient with the same or even higher energy content [38, 39], and may thus not achieve the overall goal of reducing energy density. Gressier et al. showed that reformulation strategies generally resulted in products with the same energy content and are hence unlikely to contribute to a lower energy intake in the population [38]. Since simultaneous reduction in energy content is not always easy to achieve due to technological challenges, it is even more important that reformulation strategies focus on nutritionally favourable products overall, e.g. by increasing the proportion of wholegrain in breakfast cereals.

The food market is characterised by a rapidly changing variety of products with a high proportion of new products. Thus, not only reformulation of existing products but also the launch of new products with a more favourable nutrient composition can help shift the food offer towards healthier choices. However, from a public health perspective this is only effective, if consumers switch to these products. Especially if the improvement of the overall nutritional composition of existing products (e.g. best-selling products) remains at a level as low as shown for this sample, there will be little public health benefit. Connecting product monitoring data with specific sales and consumption data on the single product level would be necessary in order to draw concrete conclusions about the public health impact of specific product reformulation or of general changes within the market portfolio. Monitoring of paired products over time is essential for assessing the contribution of reformulation to an overall improved nutritional composition of the food supply.

### Strengths and limitations

Our study sheds light on several nutrition policy issues. We were able not only to show differences between single nutrients over time in breakfast cereals on the German market but also to compare the Nutri-Score classification, including possible shifts therein due to product reformulation. A strength of the study is the combined investigation of the overall market and the subgroup of paired products. This allows for concrete insights regarding nutritional changes on the overall market as well as for reformulated products [39]. However, no clear conclusion could be drawn regarding the public health impact of the observed change in nutritional composition of the breakfast cereal supply, as the data could not be linked to sales and/or consumption data. Also, thorough as our approach for pairing products from different survey years was, we may have missed products where a change in name or presentation obscured the link.

Although the survey method was the same in both survey years, the large differences in the sample size and composition could, beside a changed market portfolio, also be due to the fact that products became more easily accessible via the internet as a result of the Covid-19 pandemic, during which online retail got more important [40].

The current investigation is based on the mandatory declared nutritional information. While we were able to draw on a large sample of breakfast cereals available on the German market, our analysis relies on the precision of the data presented on these labels or on the manufacturer’s website. It is presumed that manufacturers have adhered to EU regulations by supplying precise and up-to-date information. We carried out extensive plausibility checks to minimise erroneous data.

For products lacking specific details on the fruit content, estimations were necessary for computing the FNS. These estimations were made by a trained nutritionist on the basis of comparable products for which sufficient information was available. Therefore, it is assumed that any bias resulting from the estimation was kept to a minimum.

## Conclusion

This study showed a reduction in sugar of over 25% in children’s breakfast cereals on the German market between 2019 and 2022, along with a significant improvement of the FNS. Since there were hardly any specific product reformulations, particularly ones that led to an improved Nutri-Score, it can be inferred that changes in the overall sample were mostly driven by shifts in the product portfolio. Unless improvements in nutrient content and FNS are reflected in widely purchased and consumed breakfast cereals, benefit for consumer health will remain limited. Reformulation guided by holistic product monitoring approaches and FoPLs like the Nutri-Score can be seen as synergistic public health tools to improve the nutritional quality of the packaged food supply and enable consumers to make nutritionally more favourable food choices. Using these tools to their fullest potential appears a worthy ambition towards the goal of reducing the prevalence of overweight and obesity and with it the burden on health care systems and people’s health.

## Electronic Supplementary Material

Below is the link to the electronic supplementary material.

Supplementary Material 1

## Data Availability

The datasets used and analysed during the current study are available from the corresponding author on reasonable request.

## Abbreviations

FNS: Final nutritional score
FoPL(s): Front-of-pack label(s)
GTIN: Global trade item number
