# Supplementary material for: Using the Nutri-Score to visualise food reformulation in Germany: the case of breakfast cereals
Source: BMC Public Health. 2025 Jan 4;25:36. doi: 10.1186/s12889-024-21102-7 (PMC11699633; doi:10.1186/s12889-024-21102-7)
Supplement: Supplementary file 1 — Supplementary Material 1 [file 12889_2024_21102_MOESM1_ESM.docx]

Supplement File 1

Table S1: Mean energy and nutrient contents of breakfast cereals surveyed in Germany in 2019 (n=888) and corresponding changes in 2022 (n=1473) divided into children’s and non-children’s breakfast cereals and each category a) muesli, b) flakes, c) other cereals.

|  | **non-children‘s** | | | | **children‘s** | | | |
| --- | --- | --- | --- | --- | --- | --- | --- | --- |
| 1. **Muesli** | mean in  2019 (n^1^=1668/566) | mean change in  2022 (n^1^=1087/958) | | | mean in 2019 (n^1^=19/19) | mean change in  2022 (n^1^=114/106) | | |
|  |  | abs. | rel. (%) | p-Value |  | abs. | rel. (%) | p-Value |
| Energy [kcal/100 g] | 403 | +0.8 | +0.2 | 0.886 | 377 | **+18.4** | **+4.9** | 0.015 |
| Fat [g/100 g] | 11.5 | +0.8 | +6.7 | 0.084 | 7.0 | **+2.7** | **+39.0** | 0.016 |
| Saturated Fat [g/100 g] | 3.3 | 0.0 | -1.2 | 0.214 | 1.9 | **+0.9** | **+48.5** | 0.042 |
| Carbohydrate [g/100 g] | 59.0 | **-2.7** | **-4.6** | < 0.001 | 63.5 | **-2.0** | **-3.2** | 0.021 |
| Sugar [g/100 g] | 15.7 | **-1.4** | **-9.2** | < 0.001 | 13.5 | -0.3 | -2.3 | 0.857 |
| Protein [g/100 g] | 11.2 | **+1.1** | **+9.9** | < 0.001 | 10.9 | +0.1 | +1.0 | 0.905 |
| Salt [g/100 g] | 0.17 | **-0.02** | **-12.8** | 0.002 | 0.10 | -0.02 | -17.7 | 0.745 |
| Fibre [g/100 g] | 9.1 | **+0.5** | **+5.2** | 0.011 | 8.1 | **+0.7** | **+8.5** | 0.04 |
| Final nutritional score^2^ (FNS) | 4.5 | -0.3 | -7.1 | 0.431 | 1.0 | +1.8 | +179.3 | 0.407 |
| Bold font indicates statistically significant change in mean content in 2022 compared to 2019; p-values <0.05 were considered as statistically significant  ^1^Sample size differs, as the fibre content was not available for all products (sample size Big 7/sample size fibre content).  ^2^Sample size for FNS = 956, as the fibre content and/or ingredient list was not available to calculate the FNS | | | | | | | | |

| 1. **Flakes** | **non-children‘s** | | | | **children‘s** | | | |
| --- | --- | --- | --- | --- | --- | --- | --- | --- |
|  | mean in 2019 (n^1^=45/30) | mean change in  2022 (n^1^=67/53) | | | mean in 2019 (n^1^=9/7) | mean change in  2022 (n^1^=11/10) | | |
|  |  | abs. | rel. (%) | p-Value |  | abs. | rel. (%) | p-Value |
| Energy [kcal/100 g] | 376 | +3.6 | +1.0 | 0.814 | 376 | +1.6 | +0.4 | 0.909 |
| Fat [g/100 g] | 1.6 | **+1.6** | **+99.5** | 0.031 | 0.7 | **+1.1** | **+149.3** | 0.016 |
| Saturated Fat [g/100 g] | 0.3 | +0.9 | +286.3 | 0.246 | 0.2 | +0.2 | +157.1 | 0.196 |
| Carbohydrate [g/100 g] | 79.8 | **-4.0** | **-5.0** | 0.029 | 84.6 | -3.6 | -4.2 | 0.094 |
| Sugar [g/100 g] | 8.5 | **-2.2** | **-25.9** | 0.032 | 20.5 | -7.8 | -38.1 | 0.128 |
| Protein [g/100 g] | 8.3 | +0.6 | +7.4 | 0.363 | 6.4 | +1.1 | +16.4 | 0.128 |
| Salt [g/100 g] | 1.11 | **-0.30** | **-26.7** | 0.024 | 1.04 | -0.37 | -35.2 | 0.223 |
| Fibre [g/100 g] | 5.0 | +0.9 | +18.3 | 0.097 | 3.0 | +1.2 | +40.0 | 0.203 |
| Final nutritional score (FNS) | 7.3 | **-2.3** | **-31.2** | 0.064 | 12.9 | -6.6 | -51.0 | 0.056 |
| Bold font indicates statistically significant change in mean content in 2022 compared to 2019; p-values <0.05 were considered as statistically significant  ^1^Sample size differs, as the fibre content was not available for all products (sample size Big 7/sample size fibre content resp. FNS). | | | | | | | | |

| 1. **Other cereals** | **non-children‘s** | | | | **children‘s** | | | |
| --- | --- | --- | --- | --- | --- | --- | --- | --- |
|  | mean in 2019 (n^1^=48/42) | mean change in  2022 (n^1^=94/83) | | | mean in 2019 (n^1^=99/72) | mean change in  2022 (n^1^=100/87) | | |
|  |  | abs. | rel. (%) | p-Value |  | abs. | rel. (%) | p-Value |
| Energy [kcal/100 g] | 387 | +1.0 | +0.3 | 0.128 | 394 | +1.0 | +0.3 | 0.128 |
| Fat [g/100 g] | 5.3 | -0.6 | -10.2 | 0.417 | 5.5 | -0.6 | -10.2 | 0.417 |
| Saturated Fat [g/100 g] | 1.6 | 0.0 | -0.1 | 0.540 | 1.6 | 0.0 | -0.1 | 0.540 |
| Carbohydrate [g/100 g] | 71.1 | -0.5 | -0.7 | 0.769 | 75.0 | -0.5 | -0.7 | 0.769 |
| Sugar [g/100 g] | 20.7 | **-3.1** | **-12.4** | 0.003 | 25.0 | **-3.1** | **-12.4** | 0.003 |
| Protein [g/100 g] | 10.0 | -0.1 | -1.7 | 0.436 | 8.1 | -0.1 | -1.7 | 0.436 |
| Salt [g/100 g] | 0.43 | -0.08 | -18.0 | 0.054 | 0.44 | -0.08 | -18.0 | 0.054 |
| Fibre [g/100 g] | 7.7 | +0.4 | +6.6 | 0.379 | 5.9 | +0.4 | +6.6 | 0.379 |
| Final nutritional score (FNS) | 6.8 | -1.5 | -14.0 | 0.098 | 10.3 | -1.5 | -14.0 | 0.098 |
| Bold font indicates statistically significant change in mean content in 2022 compared to 2019; p-values <0.05 were considered as statistically significant  ^1^Sample size differs, as the fibre content was not available for all products (sample size Big 7/sample size fibre content resp. FNS). | | | | | | | | |
